# Why we need treatment? Prognosis of tricuspid regurgitation

**DOI:** 10.1093/eurheartjsupp/suaf093

**Published:** 2025-12-26

**Authors:** Josè Luis Zamorano, Pieter van der Bijl, Ariana González Gómez, Covadonga Fernández-Golfìn, Giorgia Benzoni, Maurizio Taramasso, Angel Sanchez-Recalde, Jeroen J Bax

**Affiliations:** Cardiology Department, University Hospital Ramón y Cajal, Carretera de Colmenar Km 9.100, Madrid 28034, Spain; Instituto Ramón y Cajal de Investigación Sanitaria (IRYCIS), Ctra. Colmenar Viejo, Fuencarral-El Pardo, Madrid 28034, Spain; CIBERCV, Instituto de Salud Carlos III (ISCIII), Av. Monforte de Lemos, 3-5. Pabellón 11, Planta 0, Madrid 28029, Spain; Alcalá University, Pl. de San Diego, s/n, 28801 Alcalá de Henares, Madrid, Spain; Department of Cardiology, Leiden University Medical Center, Albinusdreef 2, Leiden 2333 ZG, Netherlands; Cardiology Department, University Hospital Ramón y Cajal, Carretera de Colmenar Km 9.100, Madrid 28034, Spain; Instituto Ramón y Cajal de Investigación Sanitaria (IRYCIS), Ctra. Colmenar Viejo, Fuencarral-El Pardo, Madrid 28034, Spain; CIBERCV, Instituto de Salud Carlos III (ISCIII), Av. Monforte de Lemos, 3-5. Pabellón 11, Planta 0, Madrid 28029, Spain; Cardiology Department, University Hospital Ramón y Cajal, Carretera de Colmenar Km 9.100, Madrid 28034, Spain; Instituto Ramón y Cajal de Investigación Sanitaria (IRYCIS), Ctra. Colmenar Viejo, Fuencarral-El Pardo, Madrid 28034, Spain; CIBERCV, Instituto de Salud Carlos III (ISCIII), Av. Monforte de Lemos, 3-5. Pabellón 11, Planta 0, Madrid 28029, Spain; Cardiology Department, University Hospital Ramón y Cajal, Carretera de Colmenar Km 9.100, Madrid 28034, Spain; Department of Medicine and Surgery, University of Milano-Bicocca, Piazza dell’Ateneo Nuovo 1, Milan 20126, Italy; Heart Center Hirslanden Zurich, Witellikerstrasse, Zurich 36CH-8008, Switzerland; Cardiology Department, University Hospital Ramón y Cajal, Carretera de Colmenar Km 9.100, Madrid 28034, Spain; Instituto Ramón y Cajal de Investigación Sanitaria (IRYCIS), Ctra. Colmenar Viejo, Fuencarral-El Pardo, Madrid 28034, Spain; CIBERCV, Instituto de Salud Carlos III (ISCIII), Av. Monforte de Lemos, 3-5. Pabellón 11, Planta 0, Madrid 28029, Spain; Department of Cardiology, Leiden University Medical Center, Albinusdreef 2, Leiden 2333 ZG, Netherlands

**Keywords:** Triscupid regurgitation, Prognosis, Surgery, Transcatheter treatment, Right ventricle, Medical therapy

## Abstract

Tricuspid regurgitation (TR) has historically been under-recognized in clinical practice, yet growing evidence highlights its significant impact on prognosis, particularly in its severe forms. Severe TR is indeed associated with increased mortality and heart failure hospitalizations, with prognostic deterioration further stratified by emerging classifications such as ‘massive’ and ‘torrential’ TR. Accurate assessment of TR severity is essential for timely referral and management decisions. Traditional echocardiographic parameters—such as tricuspid annular plane systolic excursion and right ventricular (RV) fractional area change—are limited by their load-dependence, prompting growing interest in advanced imaging modalities such as strain imaging and cardiac magnetic resonance for more precise evaluation of RV function. In this setting, medical management remains only supportive, with diuretics and neurohormonal modulation forming the cornerstone of therapy, especially in patients with heart failure. However, evidence for pharmacological interventions specific to TR is limited. Surgical treatment is indicated in select patients, though associated with high perioperative risk, necessitating careful patient selection. In recent years, transcatheter tricuspid valve interventions have emerged as promising alternatives for high-risk patients, including edge-to-edge repair (T-TEER) and orthotopic tricuspid valve replacement, and also caval valve implantation is being explored for anatomically complex or high-risk cases. Early recognition, comprehensive risk assessment, and individualized therapeutic planning—including consideration of timely intervention—are crucial to improving outcomes in this often-neglected valvular condition.

## Graphical Abstract

**Graphical Abstract:**
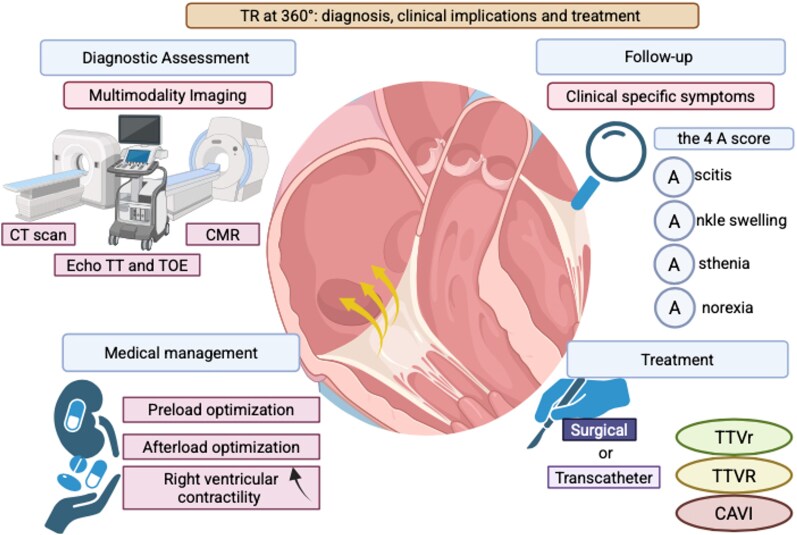
Abbreviations: TR, tricuspid regurgitation; Echo TT, transthoracic echocardiography; TOE, transesophagoeal echocardiography; CT scan, computed tomography scan; CMR, cardiac magnetic resonance; TTVr, transcatheter tricuspid valve repair; TTVR, transcatheter tricuspid valve replacement; CAVI, caval valve implantation

## Introduction

**Figure 1:**
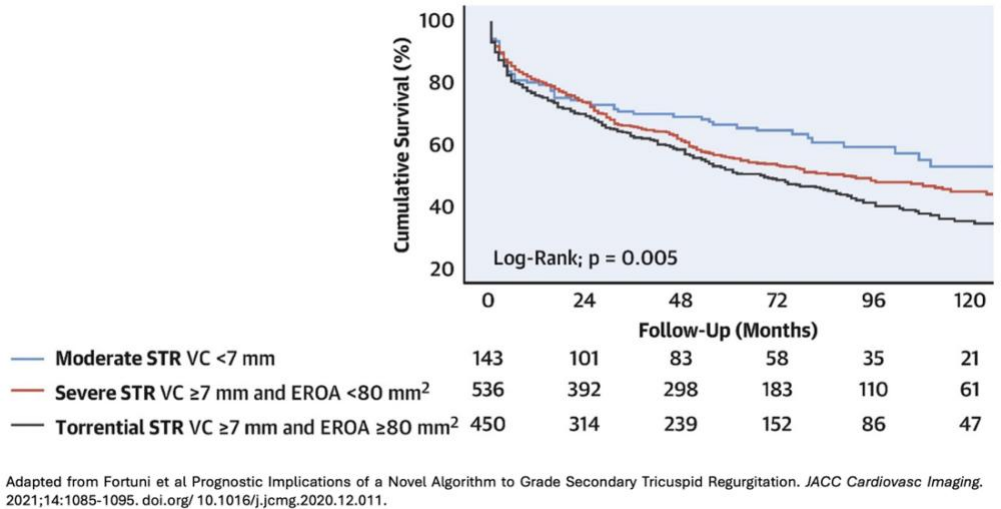
Survival of patients with tricuspid regurgitation, stratified according to severity. Abbreviation: STR, secondary tricuspid regurgitation.

The prognosis of tricuspid regurgitation (TR) is closely linked to its severity. Mild TR is, in general, tolerated well for years, while at the other end of the spectrum, severe TR is associated with increased mortality. In a study of >5000 patients, those with severe TR had only a 64% 1-year survival rate, compared to 92% in individuals with no or mild TR.^1^ In addition to mortality, moderate and severe TR increases heart failure hospitalization.^2^ Although not incorporated into guidelines yet, grades of TR beyond severe have been introduced in order to refine the risk stratification of such patients. Massive and torrential TR are defined by the vena contracta width and effective regurgitant orifice area (*Table 1*) of the TR jet.^3,4^ Torrential TR is associated with worse mortality and increased heart failure hospitalization, compared to severe TR.^3,5^ In a study of >1000 patients with secondary TR, those with torrential TR had a 10-year survival rate of 35%, compared to 45% for individuals with severe TR (*Figure 1*).^3^ Massive TR however, appears to have a prognosis similar to severe TR.^3^

**Table 1:** Echocardiographic thresholds values of tricuspid regurgitation (TR) severity quantitative parameters

Severity of TR	VC (mm)	EROA (mm^2^)
Mild	<3	<20
Moderate	3–6.9	20–39
Severe	7–13	40–59
Massive	14–20	60–79
Torrential	≥21	≥80

Abbreviations: EROA, effective regurgitant orifice area; VC, vena contracta.

In addition to the severity of TR, pulmonary hypertension, right ventricular (RV) dysfunction, renal impairment, as well as age, have an impact on prognosis. Pulmonary hypertension, while often contributing to the development and/or progression of TR, also worsens prognosis.^6^ In a study of >200 patients with moderate or severe TR, the presence of pulmonary hypertension was independently associated with all-cause mortality.^6^ Interestingly, pre-capillary pulmonary hypertension appears to portend a worse outcome than post-capillary pulmonary hypertension in the presence of TR.^5^

**Figure 2:**
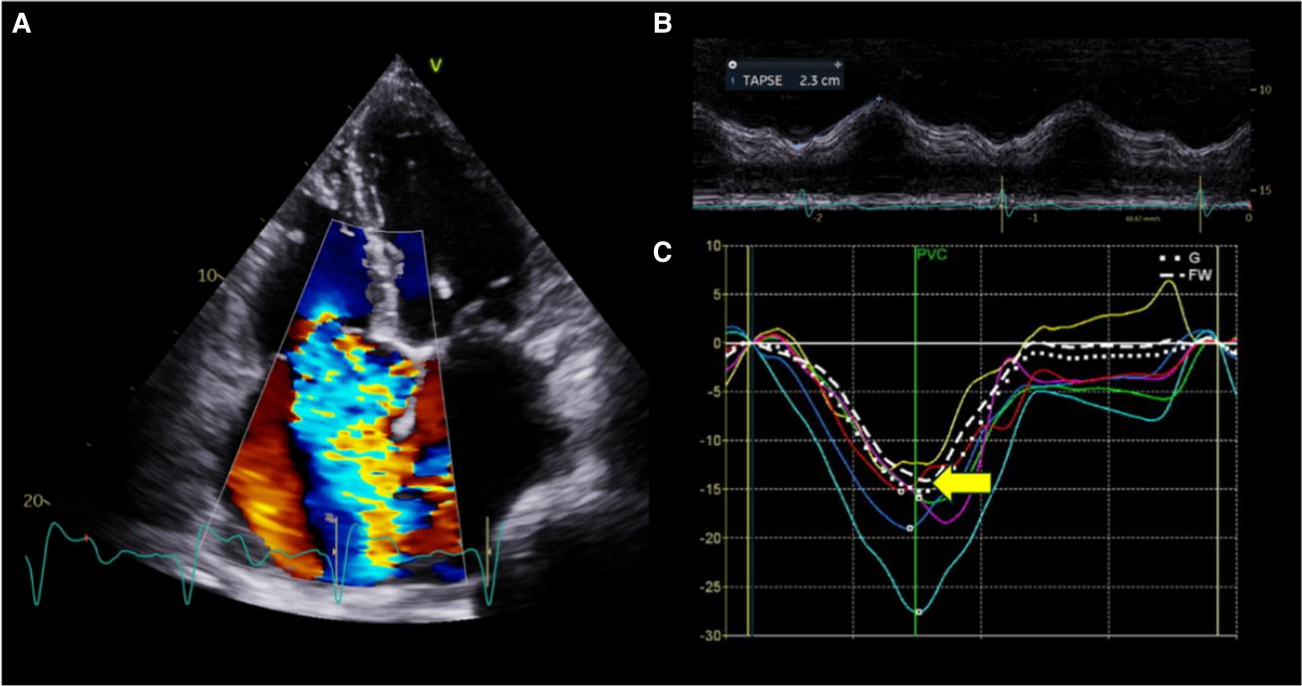
Impaired right ventricular function in a patient with severe tricuspid regurgitation (*A*). Tricuspid annular plane systolic excursion (TAPSE) is measured from an M-mode trace of the tricuspid annular motion vs. time. A TAPSE of 23 mm indicates normal right ventricular function, but may be subject to over-estimation in the presence of severe TR (*B*). Right ventricular free wall strain is reduced (−14%; yellow arrow), indicating impaired right ventricular function (*C*). Abbreviations: G, global; FW, free wall.

Severe TR causes RV volume overload and systolic dysfunction. Conversely, RV volume overload can be the proximate cause of severe, secondary TR by means of ventricular and/or tricuspid annular dilatation. Impaired RV systolic function, measured by tricuspid annular plane systolic excursion (TAPSE), was linked to increased all-cause mortality in a cohort of >1200 patients analysed by Dietz *et al.*^7^ (*Figure 2*). In a study of 539 patients with moderate or severe TR after left-sided valve surgery, RV fractional area change was independently linked to survival.^8^ The load-dependent nature of both TAPSE and RV fractional area change however, causes these parameters to over-estimate RV systolic function. In addition, the complex, three-dimensional shape of the RV makes echocardiographic function assessment challenging. Strain imaging has emerged as an alternative tool to quantify RV function in the presence of significant TR, offering a more sensitive index of RV dysfunction and incremental prognostic value over TAPSE and RV fractional area change. In a study of patients with severe TR, impaired RV free wall strain (*Figure 2*) was associated with an almost six-fold increase in mortality and heart failure, even in the presence of a normal TAPSE.^9^ RV deformation has also been shown to be a better prognosticator than TAPSE or fractional area change.^9,10^ Although not load-independent, cardiac magnetic resonance (CMR) imaging can more accurately delineate the RV volume in systole and diastole than echocardiography. In a cohort of >300 patients with severe TR, RV end-diastolic volume and RV ejection fraction, assessed by CMR, were independently correlated with heart failure and all-cause mortality.^11^ Adjusted spline models indicated that individuals with RV end-diastolic volumes >150 mL/m^2^ and a RV ejection fraction <50% were at the highest risk of heart failure and death.^11^

Renal impairment is another predictor of mortality in patients with severe TR.^12,13^ This may reflect shared risk factors or the impact of TR on renal function due to venous congestion.^12^ Older patients with TR suffer higher mortality rates.^12^ In a study of >270 patients with severe TR, age was independently associated with increased all-cause mortality.^12^

In addition to the severity of TR and the presence of comorbidities, differences in outcome have been investigated based on the aetiology of tricuspid valve dysfunction. Both TR in patients with left-sided heart disease and isolated TR (i.e. occurring in the absence of left-sided heart disease or pulmonary hypertension) are associated with increased mortality.^14,15^ In a study which included >9000 patients with moderate or severe, isolated TR, secondary isolated TR had a worse outcome than primary isolated TR.^14^ This difference, however, disappeared on multivariable analysis.^14^

## Medical treatment of tricuspid regurgitation

TR symptoms are related to the degree of volume overload and RV function. End stage of the disease is characterized by a right heart failure syndrome where the RV is not capable of generating enough stroke volume resulting in systemic venous congestion, underfilling of the left ventricle and, in late stages, low cardiac output.^16^ Although TR treatment itself is the cornerstone of patient management, this is not always possible and medical treatment to improve clinical status and quality of life is needed. In this scenario, medical treatment both in the acute and chronic settings relies on preload optimization, afterload reduction, and an increase in RV contractility. Specific features of the different types of TR should be considered as well to better adjust final treatment and management.

### Preload optimization

Although the Frank–Starling mechanism is preserved in the failing RV myocardium,^17^ the RV has a much flatter Frank–Starling curve and RV contractility changes less over a wide range of filling pressures. In TR patients, preload is usually increased, and diuretic treatment, along with ultrafiltration in severe acute cases, is needed. With preload reduction, there is an RV size and wall stress reduction, enhancing RV contractility. Dietary sodium and water intake are recommended along with loop diuretics to achieve a euvolemic state and relieve systemic congestion. Mineralocorticoid antagonists as a diuretic agent have a synergic effect and are useful for congestive symptoms.^18^

### Afterload optimization

Along with an increased preload, some types of TR patients may also have increased vascular resistance, which is a contraindication for tricuspid repair or replacement. In this situation, especially in the acute setting, general measures to correct a situation that may increase vascular resistance are recommended. Hypoxemia, acidosis, and hypercapnia should be avoided, and lung protective ventilation used if needed. Pulmonary vasodilators are currently not approved for use in critically ill patients with RV failure not due to PAH. Off-label, inhaled nitric oxide is the vasodilator of choice in critically ill patients with right heart failure and increased vascular resistance, and has been shown to improve pulmonary hemodynamic.^19^

In stable patients, no specific pharmacological drugs have demonstrated any beneficial effect on clinical status or prognosis in patients with HF and TR. However, small experimental studies have shown a potential benefit of a mineralocorticoid receptor antagonist in reducing RV afterload.^20,21^

### Increase in right ventricular contractility

An increase in RV contractility may be needed in critically ill patients. General measures should focus on avoiding overstretching of the RV wall by means of preload and afterload optimization, maintaining RV perfusion, and reducing wall stress, preserving diastolic coronary pressure. Inotropic agents may be used as well to increase RV contractility; however, robust evidence of the use of the different inotropic agents in TR patients with advanced HF is lacking. Low-dose dobutamine has been shown in animal models to restore RV-pulmonary artery coupling and increase cardiac output.^22^ Low-dose dopamine (<16 micrograms/kg/min) increases RV contractility and is especially useful in hypotensive patients. Milrinone, a selective PDE3 inhibitor, slows intracellular cyclic adenosine monophosphate (cAMP) metabolism and improves inotropy and facilitates pulmonary vasodilation.^23^ Epinephrine and norepinephrine have been used as well and are preferable in hypotensive patients. Levosimendan, a calcium sensitizer, has been shown to improve RV function in left heart disease, but didn’t prove to be superior to milrinone in cardiac surgery patients.

In the non-acute setting, no specific pharmacological treatment has proved to be useful neither in terms of clinical improvement nor in terms of mortality benefit. Only a small observational study showed a possible association between sacubitril/valsartan and improvement in RV function in patients with HF and TR.^24^

## Specific therapies

### Ventricular functional tricuspid regurgitation

Medical treatment in cases of ventricular functional TR should be based on the underlying aetiology.^18^ Therefore, patients with left ventricular dysfunction and heart failure need to be under guideline-recommended medical treatment, including beta-blockers, IECAS/ARA II/ARNIS, mineralocorticoid receptor antagonist, and iSGLT2.^25^ For those with heart failure and preserved ejection fraction, iSGLT2 along with diuretic therapy is the treatment of choice. Moreover, in a small randomized controlled trial including patients with HFrEF, sodium-glucose cotransporter-2 inhibitors in addition to other HF drugs, was found to be more effective in improving RV function as compared to other HF drugs alone.^26^

Since TR severity may change according to volemic status, reassessment of TR should be made when optimal medical therapy has been reached to avoid unnecessary interventions in those cases of improvement of TR severity.^18^ Along with medical treatment, in this clinical scenario, two interventions have been proven useful in reducing TR degree: chronic resynchronization therapy (CRT) and mitral regurgitation treatment. Reduction of TR from moderate-severe to mild has been reported up to 41% of patients after 6 months of CRT implantation.^27^ Along with TR reduction, a positive RV remodelling was also noted in these patients. In patients with LV dysfunction and moderate-severe MR and TR, edge-to-edge repair of the MR was associated with a reduction in TR severity in 35% of cases.^28^ However, the prediction criteria of positive response to either CRT or mitral valve repair area lacking.

### Atrial fibrillation

The ideal treatment for atrial fibrillation (AF) associated functional atrial TR remains unknown. Widely accepted rate control-only strategy may be associated with a higher risk of developing A-STR compared with rhythm control^29^ and the use of a rhythm control strategy in patients with early-stage paroxysmal AF to maintain sinus rhythm was associated with a lower risk of development of A-STR over a 13.3-year follow-up.^30^ Moreover, active restoration of sinus rhythm in cases of recent onset of AF was associated with a decrease in the severity of TR at 1 year due to the favourable positive remodelling of both right atrium and RV.^31^

### Lead related tricuspid regurgitation

The increasing number of electronic devices with leads through the tricuspid valve is increasing worldwide.^32^ Although preventive measures based on TEE imaging during the implantation have proved useful to reduce lead-associated TR, they are rarely used in clinical practice. Treatment strategies in this scenario include medical treatment, lead extraction, or tricuspid valve repair or replacement (more often). Medical treatment is not different from the one used in other types of TR with heart failure. Although lead extraction may be considered and seems to be safe, it is not associated with a significant reduction of TR and may produce TV harm, which may limit possible interventional treatment.^33^ Clinical decision must be individualized and discussed in this situation.

Lead related TR has also been associated with permanent RV pacing and the ongoing LV and/or RV remodelling that takes place in some patients. Accordingly, a physiological RV stimulation, or even biventricular pacing, should be considered in cases where TR is already present before implant or additional risk factors for TR development are present.^32^ However, there is no robust evidence that such an approach always translates into a decrease in TR development and clinical benefit.

## Early recognition of symptomatic status

Despite our better understanding and knowledge of the TV and the exponential growth of different interventional therapeutic options, late presentation in the course of the disease is frequently seen. This is in part due to the vague and non-specific symptoms seen in early phases of the disease and our inability to detect those symptoms with specific questions and specific scales. This way, NYHA class is used in TR patients, when it was designed and validated for left heart failure patients, which is not the problem in TR patients. Recently, Gonzalez-Gomez *et al.*^33^ designed and validated a specific classification to assess the clinical status of TR patients, the 4 A classification that proved to have superior prognosis discriminative power than NYHA class. It is based on the presence of congestive and low output symptoms (abdominal pain or distension, ankle swelling, asthenia, and anorexia) and can be easily performed in everyday clinical practice. Early detection of TR symptoms and a better definition of the clinical status will allow medical treatment instauration and prompt referral if indicated to intervention.

## Surgical treatment

The 2021 European Society of Cardiology clinical practice guidelines recommend surgical intervention in patients with severe TR (Class I) or annular dilatation (Class IIa) undergoing left-sided valve surgery, as well as in those with isolated severe primary TR (Class I).^34^ Additionally, surgery is also recommended for patients with isolated severe secondary TR who are symptomatic or with RV dilatation (Class IIa).^34^ Despite these recommendations, in-hospital mortality risk remains high for isolated tricuspid valve surgery, around 8–10%, with no significant improvement observed over time, even as procedural volumes increase.^35^ Mortality rates, however, vary significantly depending on patient profiles and the stage of disease, highlighting the importance of timely and accurate patient selection.^35,36^ The TRI-SCORE risk model integrates clinical, laboratory, and echocardiographic parameters and has demonstrated strong predictive value for both in-hospital mortality and long-term survival. Importantly, the TRI-SCORE has proven useful in identifying low-risk patients who are most likely to benefit from surgery, with reported survival rates exceeding 90% at 2 years and 70% at 10 years, clearly superior to outcomes with medical therapy alone.^37-41^ In contrast, patients categorized as high-risk by the TRI-SCORE and undergoing surgery show markedly lower survival rates, ∼20% at 10 years, and may derive no clinical benefit or even experience harm compared to conservative management.^41^

## Transcatheter tricuspid therapy

Transcatheter therapy has emerged in recent years as the therapy of choice, particularly for patients at high surgical risk or those with an intermediate or high TRI-SCORE, and encompasses two principal modalities: valve repair and valve replacement. The choice between these modalities depends on multiple clinical and anatomical factors. In general, patients with small coaptation gaps, mild leaflet tenting, RV dysfunction, or contraindication to anticoagulation are better candidates for repair techniques. Conversely, patients with torrential TR, large gaps, multiple leaflets, tethered or retracted leaflets, and lead-induced TR are more suitable for orthotopic valve replacement. Nevertheless, the final therapeutic decision must be individualized and taken in heart team^42^ (*Figure 5*).

**Figure 3:**
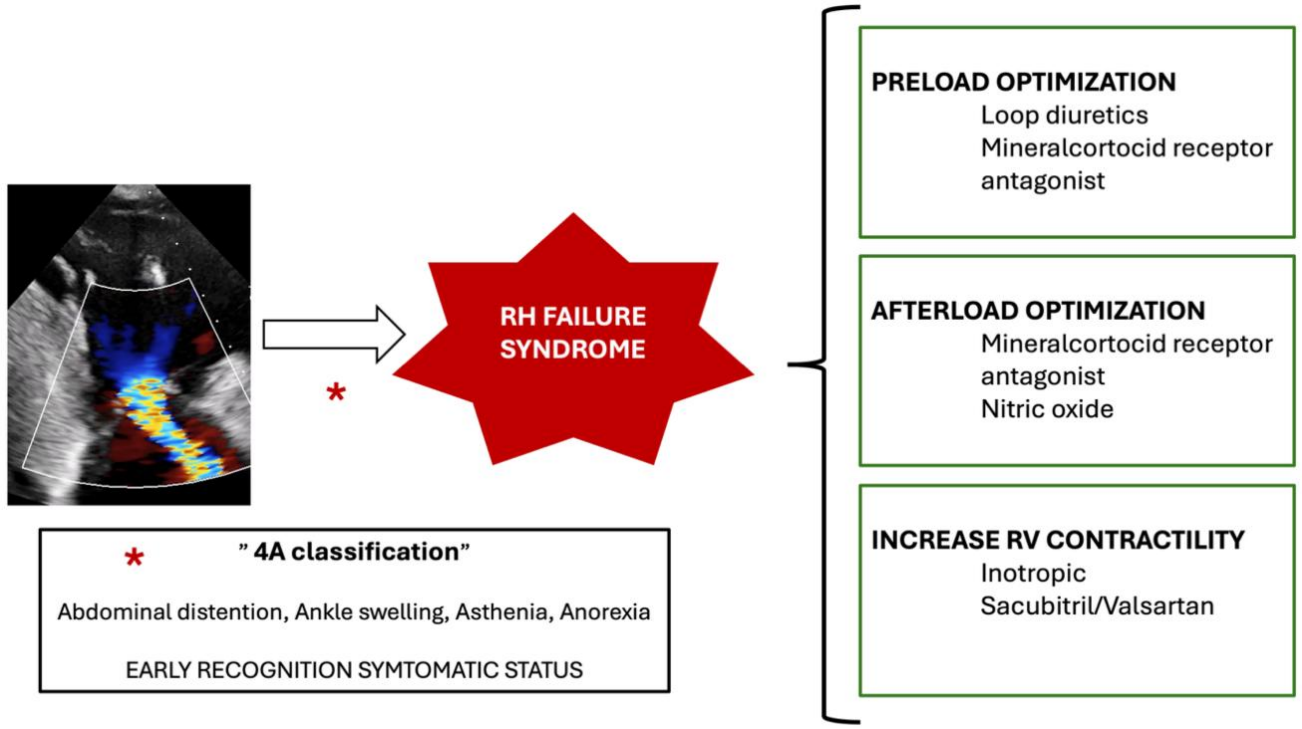
Medical treatment options in patients with tricuspid regurgitation and right heart failure symptoms. Abbreviations: RH, right heart; RV, right ventricle.

**Figure 4:**
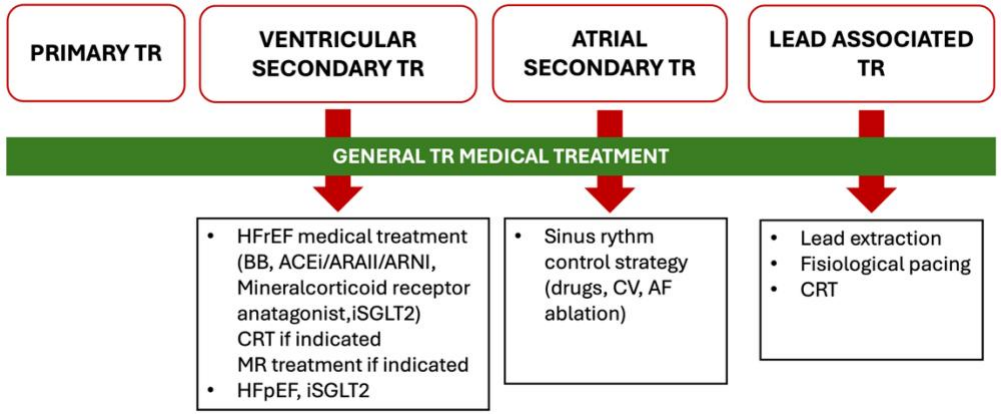
Specific treatment options according to tricuspid regurgitation mechanism. Abbreviations: TR, tricuspid regurgitation; HFrEF, heart failure reduced ejection fraction; BB, beta-blockers; ACEi, angiotensin-converting enzyme inhibitor; ARAII, Angiotensin II receptor antagonist; ARNI, angiotensin receptor–neprilysin inhibitor; iSGLT2, sodium–glucose cotransporter 2 inhibitor; CRT, cardiac resynchronization therapy; MR, mitral regurgitation; HFpEF, heart failure preserved ejection fraction; CV, cardioversion; AF, atrial fibrillation.

**Figure 5:**
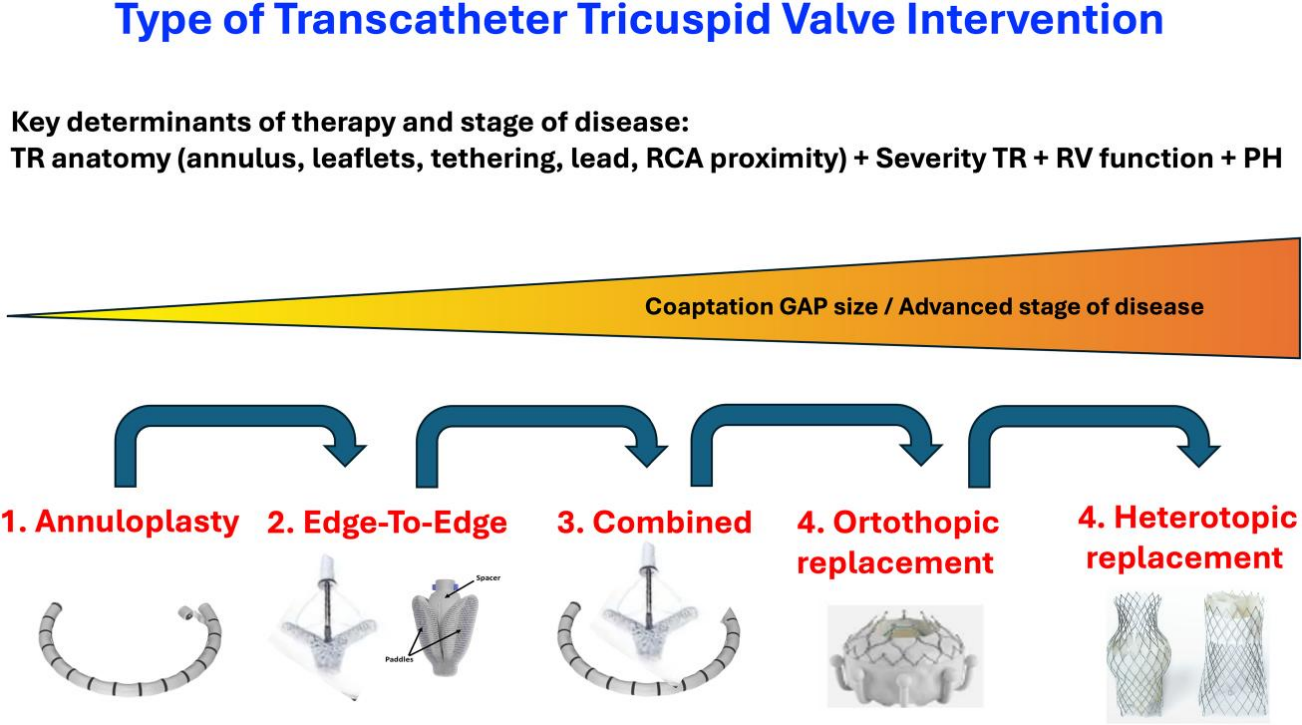
Different types of transcatheter tricuspid valve intervention according to the stage of the tricuspid valve disease. Abbreviations: PH, pulmonary hypertension; RCA, right coronary artery; RV, right ventricle; TR, tricuspid regurgitation.

### Tricuspid valve repair

Several transcatheter repair strategies have been developed, with annuloplasty and leaflet approximation representing the two principal approaches. Annuloplasty is considered the most physiological technique, especially in patients with atrial secondary TR, where annular dilation is the predominant pathophysiological mechanism. The Cardioband system (Edwards Lifesciences) was the first annuloplasty device to receive CE marking in 2018. It was designed to achieve direct annular reduction by cinching the non-mural annulus from the anteroseptal to the posteroseptal commissure.^43-45^ Greater annular coverage has been associated with more substantial RV reverse remodeling.^46,47^ The first-generation Cardioband device has since been discontinued. A feasibility study is currently underway for a second-generation system, which is expected to offer a simplified implantation process and incorporate a patient-specific customized band. Key pivotal studies are summarized in *Table 2*.

**Table 2:** Key trials of CE-marked transcatheter tricuspid repair devices

Study name	Design	Devices	Number of patients	Primary endpoint	TR reduction at 30 days	Post-procedural mortality	1 year mortality	Clinical outcomes/hospitalizations
**TEER**								
TRILUMINATE Study (final 3-year outcomes) (NCT03227757**)**	Prospective, single-arm, observational study	TriClip	98 (full cohort)	Primary safety endpoint, defined as a composite MAE Efficacy endpoint was reduction in TR severity by at least 1 grade	TR reduction to ≤2+: 79% at 3 years TR reduction of at least 1 grade: 92% at 3 years	6-month mortality: 5%	1-year mortality: 7.3% 2-year mortality: 18.7% 3-year mortality: 27.0%	Improvement in NYHA class, KCCQ score at 3 years Reduction in hospitalizations between the period before and after device implantation: 84% at 2 years; 75% at 3 years
CLASP TR Early Feasibility Study (NCT03745313)	Prospective, single-arm, observational study	PASCAL	65	Safety endpoints: CEC-adjudicated MAEs at 30 days; Performance endpoints (implant, procedural and clinical success); echocardiographic and clinical endpoints	At least 1 grade reduction: 100% TR reduction to ≤2+: 86%	30-day mortality: 0%	12.1 at 1 year	Improvement in NYHA class, KCCQ score, and 6MWT at 30 days HFH at 1 year: 21.5%
PASTE	Investigator-initiated, real-world, multicentre, retrospective and prospective study	PASCAL	1059	Safety and efficacy endpoints according to TVARC	TR reduction to ≤2: 87%	30-day mortality 1.3%	1-year mortality: 14%	Improvement in NYHA class, KCCQ score, and 6MWT at 30 days HFH at 30 days: 1.6% HFH at 1 year: 16%
TriValve	Prospective, multicentre, international registry	TriClip	249	All cause mortality and composite of all-cause mortality and unplanned rehospitalization for HF at 12 months	TR reduction to ≤2: 77%	In-hospital mortality: 2.8%	20.3%	Significant improvement in NYHA class Combined all-cause mortality and unplanned HFH at 1 year: 34%
bRIGHT Post-Approval Study (NCT04483089)	Prospective, post-market survey	TriClip	511	Procedural success (TR reduction of at least 1 grade); safety endpoints (MAEs); echocardiographic and clinical endpoints	TR reduction to ≤2+: 81% at 1 year	30-day mortality: 1.0%	15.1% at 1 year	Improvement in NYHA class and KCCQ score 15.3% HFH at 1 year
TRILUMINATE Pivotal Trial (NCT03904147)	Prospective randomized control trial (control arm: medical therapy alone)	TriClip	572 (full cohort)	Composite of death from any cause or TV surgery; hospitalization for HF; improvement in QoL measured with KCCQ at 1-year FU	TR reduction to ≤2+: 88.9% at 30 days; 87.6% at 1 year	30-day mortality: 0.2%	1-year all-cause mortality: 8.6% 2-year all-cause mortality: 17.9%	A significant treatment effect was observed for change in quality of life with 52.3% of device subjects achieving a≥15-point KCCQ score improvement (vs. 23.5% of control subjects; *P* < 0.0001 In first year of FU, annualized HFH rate was comparable between device and control subjects (0.17 vs. 0.20 events/patient-year; *P* = 0.40) Annualized HFH rate through 2 years was significantly lower with t-TEER compared with control (0.19 event per patient-year vs. 0.26 event per patient-year; *P* = 0.02;
TRI.FR Randomized Clinical Trial	Investigator-initiated, prospective, randomized (1:1) trial. T-TEER + OMT vs. OMT alone	TriClip	300 (152 for intervention)	Composite clinical endpoint (NYHA, change in patient global assessment, or occurrence of major cardiovascular events during 12-month period after randomization	TR reduction to ≤2 at 1 year: 78.3%	30-day mortality: 0.7%	3.4% vs. 5.4% (NS)	TEER improves the composite clinical outcome comprising NYHA class, PGA and major CV events at 12 months. Greater improvement in KCCQ score and greater increases in 6MWT distance compared to OMT Mean overall KCCQ summary score at 1 year was 69.9 (SD, 25.5) for the T-TEER + OMT group and 55.4 (SD, 28.8) for the OMT-alone group (*P* < 0.001) At 1 year, 109 patients (74.1%) in the T-TEER + OMT group had an improved composite score compared with 58 patients (40.6%) in the OMT-alone group he T-TEER + OMT group experienced greater increases in 6MWT distance (from 302 [SD, 104] to 333 [SD, 120] m, mean +10%) than the OMT-alone group (from 309 [SD, 112] to 302 [SD, 116] m, mean +2%) 1-year HFH rate: 9.9% for the T-TEER + OMT group vs. 13.5% for the OMT-alone group (NS)
**Direct annuloplasty**								
TRI-REPAIR Study (NCT02981953)	Prospective, single-arm, observational study	Cardioband	30	Performance endpoint: successful access, deployment and positioning of the device and reduction of the septolateral annular diameter Safety endpoint: rate of MSAEs and SADEs at 30 days	TR reduction to ≤2+: 76%	30-day mortality: 6.7%	17% at 1 year 27% at 2 years	Improvement in NYHA class, KCCQ score, and 6MWT at 6 months HFH at 1 year: 31% HFH at 2 years: 44%
Cardioband EFS (NCT03382457)	Prospective, single-arm, observational study	Cardioband	37	Device success, procedural success and clinical success at 30 days Reduction in TR severity and TV remodelling	TR reduction to ≤2+: 73% at 1 year	30-day mortality: 0%	14.1% at 1 year	Improvement in NYHA class and KCCQ score at 1 year HFH at 1 year: 11.3%
TriBAND Study (NCT03779490)	Prospective, post-market survey	Cardioband	61	Reduction in TR severity between baseline and discharge	TR reduction to ≤2+: 69% 89% of patients had at least one grade TR reduction	30-day mortality: 1.6%	NA	Improvement in NYHA class and KCCQ score at 30 days

Abbreviations: HFH, heart failure hospitalizations; KCCQ, Kansas City Cardiomyopathy Questionnaire; MAEs, major adverse events; NYHA, New York Heart Association; TR, tricuspid regurgitation; 6MWT, 6-min walk test

The most widely adopted transcatheter repair technique is tricuspid edge-to-edge repair (T-TEER), which enhances leaflet coaptation by grasping the tricuspid valve leaflets. The first T-TEER device to receive CE marking was the TriClip system (Abbott), approved in 2020. The latest generation—TriClip G4—offers a range of clip sizes to accommodate varying anatomical challenges. Among these, the XTW clip, which is the longest and widest option, is the most frequently used due to its ability to maximize leaflet grasping and achieve greater degrees of annular reduction. The TRILUMINATE Pivotal trial represents a landmark in this field, as it was the first randomized study to evaluate an isolated transcatheter TR intervention with Triclip against optimal medical therapy (OMT).^48^ In this trial, 350 symptomatic patients with severe TR were enrolled and randomized. The primary endpoint—a hierarchical composite assessed at 12 months—included all-cause mortality or tricuspid valve surgery, hospitalizations for heart failure, and quality-of-life improvement as quantified by the Kansas City Cardiomyopathy Questionnaire (KCCQ) > 15 points at 12 months. At 1 year, no significant differences were observed between the T-TEER and control groups in terms of mortality, tricuspid valve surgery, or heart failure hospitalization rates. However, patients treated with T-TEER demonstrated a clinically meaningful improvement in quality of life, reflected by a 12.3 ± 1.8-point increase in the KCCQ score, compared with a modest 0.6 ± 1.8-point gain in the control arm. A direct correlation was observed between the degree of TR reduction and the magnitude of quality-of-life improvement, suggesting a dose–response relationship.^49^

Notably, in the pre-specified analysis of recurrent heart failure hospitalizations at 2 years, treatment with the TriClip device resulted in a 28% relative risk reduction (0.19 vs. 0.26 events per patient-year; *P* = 0.02), despite a high rate of crossover to device therapy in the control group.^50^ Interestingly, the divergence in hospitalization rates between groups became apparent only after the first year of follow-up. Nearly 60% of patients originally assigned to the control arm ultimately crossed over to device therapy, highlighting the inadequacy of medical treatment alone in achieving sustained symptom relief and quality-of-life improvement. Following crossover, patients experienced meaningful clinical benefit. At 2 years, 84% of device-treated patients had moderate or less residual TR, and improvements in quality of life were sustained. All-cause mortality remained similar between groups (17.9% vs. 17.1%).

The TRI-FR trial is the second randomized study to T-TEER (Triclip) plus OMT vs. OMT alone in patients with severe symptomatic TR. Conducted in France and Belgium, the trial enrolled 300 patients and reported results at 1 year that were consistent with those of the TRILUMINATE pivotal study.^51^ The primary endpoint was a composite clinical outcome at 1 year, including change in New York Heart Association (NYHA) functional class, patient global assessment, and occurrence of major cardiovascular events. T-TEER combined with OMT significantly reduced TR severity and improved clinical status, primarily driven by improvements in NYHA class and patient global assessment. The composite clinical benefit was observed in 74.1% of patients in the T-TEER + OMT group, compared with only 40.6% in the OMT-alone group. No significant differences were found between groups in all-cause mortality or heart failure hospitalizations.

Taken together, the results of these two randomized trials indicate that T-TEER provides early improvements in functional capacity and quality of life, with a reduction in heart failure rehospitalizations becoming evident after the first year of follow-up, and no observed differences in mortality at 2 years. It is important to note that patients with severe RV dysfunction, pulmonary hypertension, or end-stage organ failure were excluded from these randomized trials, as the procedure is unlikely to provide clinical benefit in this population. Finally, in patients with large coaptation gaps, annuloplasty may be an attractive strategy to reduce annular dimensions and facilitate subsequent T-TEER.^52^

### Tricuspid valve replacement

For patients with advanced stages of disease, characterized by a markedly dilated tricuspid annulus and large coaptation gaps, orthotopic transcatheter tricuspid valve replacement (TTVR) may represent a more appropriate therapeutic strategy. Among the various investigational systems under evaluation, the EVOQUE valve (Edwards Lifesciences) obtained CE marking in October 2023 and is currently the only TTVR device approved for clinical use in Europe. In the TRISCEND II trial, 400 patients with symptomatic severe TR were randomized to undergo TTVR with the EVOQUE system or to receive medical therapy alone.^53^ The primary hierarchical composite endpoint included all-cause mortality, implantation of an RV assist device or heart transplantation, reintervention on the tricuspid valve, heart failure hospitalization, and clinically meaningful functional improvements—defined as a ≥10-point increase in the KCCQ-OS, ≥1 NYHA class improvement, and ≥30-m gain in the 6-min walk test. TTVR with EVOQUE demonstrated high efficacy in TR reduction, with 95.3% of patients achieving none or mild residual regurgitation, and was superior to medical therapy for the composite endpoint, largely driven by significant improvements in symptoms and health-related quality of life.^54^

However, unlike valve repair, where procedural complications are uncommon, the TRISCEND II trial reported notable device-related adverse events: new permanent pacemaker implantation in 24.7% of patients without prior pacing devices, and severe bleeding in 10.4%. Additional complications such as prosthesis malposition or migration, cardiac tamponade, and valve thrombosis have also been observed, though their true incidence will be better elucidated in post-marketing surveillance data, including the ongoing TRISCEND III registry.^55^

Caval valve implantation (CAVI) has primarily been employed in patients with anatomical contraindications to orthotopic transcatheter tricuspid interventions or in those with prior transcatheter therapy failure. Importantly, CAVI can be performed under local anaesthesia, guided by fluoroscopy and transthoracic echocardiography, and is independent of native tricuspid valve anatomy—making it a feasible option for anatomically challenging or high-risk patients. The TricValve system (Products & Features) is currently the only CAVI device with CE marking, which it received in 2021. Data from the TRICUS EURO registry demonstrated improvements in both functional capacity and quality of life following TricValve implantation.^56,57^ More recently, the TRICBICAVAL registry reported a reduction in heart failure–related hospitalizations compared with the year preceding device implantation, along with sustained improvements in functional status and quality of life. A randomized controlled trial, TRICAV II, is currently in preparation and will compare OMT alone vs. TricValve plus OMT.^58^ Key pivotal studies are summarized in *Table 3.*

**Table 3:** Key trials of CE-marked TTVR

Study name	Design	Devices	Number of patients	Primary endpoint	TR reduction at 30 days	Post-procedural mortality	1 year mortality	Clinical outcomes/hospitalizations
Tricuspid orthotopic prosthesis								
TRISCEND Study (NCT04482062)	Prospective, single-arm, observational study	EVOQUE system	176	Safety endpoints: MAEs at 30 days, procedural success, and clinical endpoints at 1 year	TR reduction to ≤1+: 97.6%	30-day cardiovascular mortality: 1.7%	All-cause mortality 9.1%	NYHA class I or II was achieved in 93% of patients (vs. 25% before intervention), the KCCQ score increased by 26 points, 6MWD increased by 56 meters, at 1 year
TRISCEND II Pivotal trial (NCT04482062)	Prospective randomized control trial (control arm: medical therapy alone)	EVOQUE system	400	All-cause death, implantation of a right ventricular assist device or heart transplantation, tricuspid-valve intervention, hospitalization for heart failure, improvement of at least 10 points in the score on the KCCQ improvement of at least one NYHA functional class, improvement of at least 30 m on the 6MWT	TR reduction to ≤1 + at 6 months: 95.3%	30-day all-cause mortality: 3.5%	All-cause mortality valve replacement 11.6% vs. 10.5% control group.	66.4% had an increase of at least 10 points in the KCCQ-OS score, 78.9% had a decrease of at least one NYHA class, and 47.6% increased their 6MWT by at least 30 m
Tricuspid Heterotopic Devices								
TRICUS EURO study (NCT04141137)	Prospective, single-arm, observational study	TRICVALVE	35	Quality-of-life improvement measured by KCCQ score and NYHA functional class improvement at 6-month follow-up	—	6-month mortality: 8.5%		Increase in KCCQ score 42 points at baseline to 59.7 at 6-month follow-up (*P* = 0.004).NYHA functional class 79.4% of patients in functional class I or II at 6 months (vs. 0% at baseline).
TRICBICAVAL Registry	Retrospective, single-arm, observational study	TRICVALVE	204	Change in NYHA functional class and systemic venous congestion at 1-year, as well as the rate of unplanned rehospitalization for heart failure within 12 months after the procedure compared to the year before the procedure	—	30-day mortality: 5.7%	All-cause mortality at 1-year: 18.6%	81.6% of patients were in NYHA class I or II, compared to 19.8% at baseline. Peripheral oedema decreasing from 73.0% at baseline to 22.1% at one year follow-up (*P* < 0.001). HF annualized hospitalization rates significantly decreased in the first year post-CAVI compared to the year prior (60.8 [95% CI 50.5–73.1] vs. 26.9 [95% CI 19.1–37.8] events per 100 patient-years; *P* < 0.001)

Abbreviations: KCCQ, Kansas City Cardiomyopathy Questionnaire; MAEs, major adverse events; NYHA, New York Heart Association; TR, tricuspid regurgitation; 6MWT, 6-min walk test.

## Conclusion

TR is a complex and increasingly recognized condition with significant prognostic implications, especially as severity progresses beyond the traditional ‘severe’ classification to massive and torrential TR. Prognosis is influenced not only by TR severity but also by RV function, pulmonary hypertension, renal function, and patient age. Medical therapy remains largely supportive, focused on symptom management through preload and afterload optimization, and improving RV function in advanced stages. Surgical intervention is limited by high operative risk in many patients, highlighting the importance of careful patient selection through tools like the TRI-SCORE.

In recent years, transcatheter therapies have significantly advanced the management of TR, offering effective options for high-risk patients. Techniques such as T-TEER, TTVR, and CAVI have demonstrated improvements in symptoms, quality of life, and, in some cases, reductions in heart failure hospitalizations. However, mortality benefits remain limited, and procedural risks—particularly with TTVR—must be weighed carefully. Early identification of TR-related symptoms, comprehensive risk stratification, and a tailored, multidisciplinary approach are essential to optimizing outcomes in this challenging patient population.

## Data Availability

The data underlying this article will be shared on reasonable request to the corresponding author.
